# Nicotinic α7 receptors on cholinergic neurons in the striatum mediate cocaine-reinforcement, but not food reward

**DOI:** 10.3389/fnmol.2024.1418686

**Published:** 2025-01-21

**Authors:** Michael Fritz, Priscila Batista Rosa, Daniel Wilhelms, Maarit Jaarola, Johan Ruud, David Engblom, Anna M. Klawonn

**Affiliations:** ^1^Department of Biomedical and Clinical Sciences, Linköping University, Linköping, Sweden; ^2^School of Health and Social Sciences, AKAD University of Applied Sciences, Stuttgart, Germany; ^3^Department for Forensic Psychiatry and Psychotherapy, Ulm University, Ulm, Germany; ^4^Department of Emergency Medicine, Linköping University Hospital, Linköping, Sweden; ^5^Department of Physiology, Institute of Neuroscience and Physiology, Sahlgrenska Academy, University of Gothenburg, Gothenburg, Sweden; ^6^Danish Institute of Translational Neuroscience (DANDRITE), Nordic EMBL Partnership for Molecular Medicine, Aarhus University, Aarhus, Denmark; ^7^Department of Biomedicine, Aarhus University, Aarhus, Denmark; ^8^Department of Psychiatry and Behavioral Sciences, Stanford University, Stanford, CA, United States

**Keywords:** α7 nicotinic acetylcholine receptor, Chrna7, striatum, cocaine, food reward, immediate early genes

## Abstract

The neurotransmitter acetylcholine has since long been implicated in reward learning and drug addiction. However, the role of specific cholinergic receptor subtypes on different neuronal populations remain elusive. Here, we studied the function of nicotinic acetylcholinergic alpha 7 receptors (α7 nAChRs) in cocaine and food-enforced behaviors. We found that global deletion of α7 nAChRs in mice attenuates cocaine seeking in a Pavlovian conditioned place preference paradigm and decreases operant responding to cocaine in a runway task and in self-administration, without influencing responding to palatable food. This effect can be attributed to alpha 7 receptor signaling in the striatum, as selective deletion of striatal α7 nAChRs using a viral vector approach resulted in a similar decrease in cocaine-preference as that of global deletion. To investigate which type of striatal neurons are responsible for this effect, we selectively targeted Cholinergic (ChAT-expressing) neurons and dopamine D1-receptor (D1R) expressing neurons. Mice with conditional deletion of α7 nAChRs in ChAT-neurons (α7 nAChR-ChATCre) exhibited decreased cocaine place preference and intact place preference for food, while α7 nAChR-D1RCre mice had no changes in reward learning to neither food nor cocaine. Cocaine induction of striatal immediate early gene expression of cFos, FosB, Arc and EGR2 was blocked in α7 nAChR-ChATCre mice, demonstrating the importance of α7 nAChRs on cholinergic neurons for striatal neuronal activity changes. Collectively, our findings show that α7 nAChRs on cholinergic interneurons in the striatum are pivotal for learning processes related to cocaine, but not food reward.

## Introduction

Several studies have implicated the neurotransmitter acetylcholine in reward learning to drugs of abuse. It was demonstrated that striatal acetylcholine is increased in rats during reward learning induced by cocaine or opioids, but not by food-reward (Crespo et al., 2006, 2008). In line with these findings, optogenetic inhibition of cholinergic interneurons in nucleus accumbens (NAc) of mice prevents acquisition of cocaine conditioned place preference (CPP; Witten et al., 2010), while activating cholinergic interneurons in the NAc during extinction promotes the loss of CPP to cocaine, without affecting food CPP (Lee et al., 2016). These findings are intriguing as they challenge the idea that all reward learning—from natural rewards to drugs of abuse—shares the same neurotransmitter basis, and essentially that drugs of abuse hijack the reward system. Collectively, these studies indicate that NAc cholinergic transmission could be a selective signaling mechanism of reward learning related to drugs of abuse, distinct from natural reward learning.

Cholinergic transmission in the mesolimbic reward system comes from different sources: The cholinergic projections from the two brainstem nuclei, laterodorsal tegmental nucleus and pedunculopontine nucleus, as well as from a small (1–2% of all neurons) striatal interneuron population (Dautan et al., 2014; Oakman et al., 1995).

Acetylcholine signals through two receptor-families, originally identified based on their pharmacological ligand selectivity: the nicotinic and the muscarinic acetylcholine receptors. Nicotinic acetylcholine receptors (nAChRs) are fast-acting, ligand-gated ion channels that allow cation diffusion upon acetylcholine binding (Mihailescu and Drucker-Colin, 2000). These receptors usually consist of an arrangement of five subunits, creating two ligand binding-sites and a central ion-pore. The nicotinic receptors are either homomeric (with α7, α8 or α9 monomers) or heteromeric (with combinations of α2–α6 and β2–β4 monomers), where the α7-receptor is the only CNS-specific homomer (Dani and Bertrand, 2007; Fowler et al., 2008; Albuquerque et al., 2009). The two most widely expressed receptor types in the mammalian CNS are the α7 homomeric and α4β2 heteromeric nAChRs (Hendrickson et al., 2013).

The α7 nicotinic acetylcholine receptors (α7 nAChRs) have properties that make them powerful excitatory effectors, which include a low affinity for agonists, fast activation, large conduction and a high Ca^2+^:Na^+^ permeability-ratio exceeding that of NMDARs (Albuquerque et al., 2009).

The role of the specific acetylcholinergic receptor populations and especially the α7 nAChR in substance use disorders has remained largely unexplored (Larsson et al., 2002; Hendrickson et al., 2009; Kuzmin et al., 2009).

Not surprisingly, most studies on the role of α7 nAChRs in drug addiction are on nicotine reward learning and withdrawal. Both the selective α7 nAChR antagonist methyllycaconitine (MLA) and α7 nAChR deletion attenuates nicotine self-administration, while MLA specifically decreases nicotine place preference (Markou and Paterson, 2001; Laviolette and van der Kooy, 2003; Walters et al., 2006; Besson et al., 2012). Furthermore, the α7 nAChRs have been implicated in nicotine withdrawal, as both ventral tegmental area (VTA) MLA injections and α7 nAChR deletion decrease the potentiating effect of nicotine on intracranial self-stimulation (ICSS) reward (Panagis et al., 2000; Stoker et al., 2012). In line with this, Salas et al. demonstrated that the somatic effects of nicotine withdrawal are significantly reduced by α7 nAChR ablation (Salas et al., 2007).

Interestingly, the α7 nAChRs have also been implicated in reinforcement to other drug-classes. It has been shown that MLA inhibits morphine CPP reinstatement (Feng et al., 2011) and decreases self-administration of cannabinoids (Solinas et al., 2007) in rats. VTA MLA injections and α7 nAChR deletion block the potentiating effect of cocaine on intracranial self-stimulation (ICSS) reward (Panagis et al., 2000). However, while α7 nAChR deletion decreases acute cocaine induced locomotion (Villégier et al., 2010), MLA does not affect cocaine induced locomotion, nor locomotor sensitization in rats when injected in the NAc or VTA (Champtiaux et al., 2006). These results prompt further investigations of the role of α7 nAChRs and the neurons they control in reward behavior using selective targeting tools.

Here, we explored the role of α7 nAChRs in cocaine and food reward learning using conditional transgenic targeting of α7 nAChRs on specific subsets of striatal neurons. We explored if global α7 nAChRs are necessary for Pavlovian and operant responding to cocaine and food; and if Pavlovian reward conditioning effects can be attributed to the striatum and specific dopamine D1 receptor (D1R) or Choline Acetyl Transferase (ChAT) expressing neuro-populations. Finally, we investigated if specific deletion of α7 nAChRs from ChAT-neurons leads to changes in striatal immediate early gene (IEG) expression after acute cocaine exposure.

## Materials and methods

### Animals

All animals in this study were male mice with a C57Bl6J background and an average age of 8–12 weeks. α7 nAChR (Chrna7) global deletion mice (RRID:IMSR_JAX:003232), α7 nAChRs (Chrna7)^floxed^ (RRID:IMSR_JAX:026965) and ChATCre mice (RRID:IMSR_JAX:006410) for breeding, were purchased from The Jackson Laboratory. α7 nAChR (Chrna7) eGFP mice were obtained from the Mutant Mouse Regional Resource Center. The D1RCre-line is described in the literature (Jeon et al., 2010). Wild-type littermate animals carrying two floxed alleles of the α7 nAChRs (WT) were used as controls for experiments using α7 nAChRs (Chrna7)^floxed^-ChATCre and α7 nAChRs (Chrna7)^floxed^-D1RCre mice.

Mice were single-housed 48 h prior to experiments, housed with environmental enrichment and kept in a pathogen-free facility on a regular 12-h light/dark cycle. All experiments were performed during the light phase. Food and water were provided *ad libitum* in the home-cage, with the exception of animals in the food-reward operant paradigms, as described below. The use of animals for this study followed the EU directive 2010/63/EU for animal experiments and were approved by the Research Animal Care and Use Committee in Linköping, Sweden.

### Drugs

Cocaine HCl was obtained commercially from Sigma-Aldrich and the Hässleholm Hospital Pharmacy, Sweden, and dissolved in saline. The dose of cocaine and administration route is described under each behavioral-test in the method-sections below. In general, mice received 15 mg/kg cocaine unless otherwise specified.

### Locomotion

Locomotion was monitored in a standardized locomotor chamber box (450 [W] × 450 [D] × 400 [H] mm) divided in 4 equal-sized compartments from Panlab, Harvard Apparatus. The locomotor activity of 4 mice was monitored simultaneously over 30 min using SMART VIDEO TRACKING software (Panlab, Harvard Apparatus). On day 0, mice received an intraperitoneal (i.p.) saline injection immediately before being placed into the tracking box. From day 1 to day 6 mice received 15 mg/kg cocaine i.p. immediately before video-tracking. Subsequently, the mice were left in their home-cages for 14 days. On day 20, the mice were placed back into the tracking box without any injection to monitor conditioned locomotor activity. On day 21, the mice received a final injection of 15 mg/kg cocaine and were video-tracked, in order to study drug-induced sensitization effects.

### Conditioned place preference

We used a place conditioning procedure to measure preference, applying a 3-chambered Panlab Spatial Place Preference Box (Harvard Apparatus), according to previously published protocols, (Klawonn et al., 2017). On day 1, during a 15-min pretest, the individual mouse was allowed to move freely between the chambers of the box. Time spent in each compartment was manually recorded by two independent experimenters blinded to genotype. To ensure explorative behavior during the pretest, each mouse had to cross the corridor, entering the opposing chamber a minimum of 5 times to be included in the experiment. Any animal that spent >66% of their time during pretests in either of the conditioning chambers were discarded from the study. Mice were assigned to vehicle- or cocaine-paired compartments in a manner to avoid reinforcing natural bias, i.e., cocaine injections were paired with the least preferred chamber identified during pretest. This method has been shown to produce reliable conditioned place preference responses to cocaine (Klawonn et al., 2017, 2018). On day 2 in the morning, mice were injected with saline i.p. before confinement for 15 min in the vehicle chamber. 4 h later on the same day, the mice were trained to cocaine (15 mg/kg) i.p. in the opposite chamber. This alternating training procedure was continued for 4 consecutive days, until day 6, when the conditioned place preference was assessed by allowing the mice to freely explore all compartments of the box for 15 min. The individual preference score (i.e., conditioning score) was calculated by subtracting the time the mouse spent in the cocaine-paired chamber during the pretest from that of the posttest. The conditioning boxes were thoroughly cleaned after each mouse using warm water and a surface disinfectant (70% isopropanol).

For natural reward place preference, we employed the same conditioning procedure as for cocaine. A small amount of Nutella (Ferrero©) on a grey tile was used as the unconditioned stimulus during training, compared to a clean grey tile in the non-conditioning chamber.

### Catheter surgery

Catheterization was performed under anesthesia induced by a mixture of 1 mg/kg dexmedetomidine and 75 mg/kg ketamine i.p. using aseptic surgical techniques. Following induction, the mice received i.p. analgesia (0.1 mg/kg buprenorphine). A catheter (MIVSA mouse catheter, CamCaths) with a 9.0-cm length of silicone tubing (inner diameter, 0.2 mm; outer diameter, 0.4 mm) was inserted 1.2 cm into the right jugular vein, tunneled s.c. to the ventral aspect of the neck, and anchored to a 26-gauge stainless steel tubing in plastic secured s.c. with a propylene knitted mesh (diameter, 20 mm). Catheter patency was confirmed by backflush, and skin incisions were closed with Prolene 6-0 (Johnson & Johnson). Anesthesia was reversed with atipamezol (1 mg/kg) s.c., and the mice were left to recover in a euthermic environment. After surgery, mice were given i.p. analgesic (buprenorphine, 0.1 mg/kg) at least every 12th hour for 2 days.

### Operant runway

For operant reinforcement, we used a custom-made mouse-runway built by AgnThos AB, according to previously published protocols, (Klawonn et al., 2018). The runway consisted of 2 chambers measuring 14 cm × 14 cm × 25 cm and equipped with retractable doors connected by a grey corridor (8 cm × 80 cm × 25 cm). The start-chamber was white, while the goal-chamber had black on white dotted wall-paper, black floor and a cue-light. For the palatable food runway, mice were habituated to Nutella® in their home-cages for 2 days and food-restricted 4 h prior to the experiment. At the start of each trial (run), a small quantity Nutella® was placed in the goal-chamber. Each mouse was given 5 consecutive runs separated by 1 h, which was spent in the home-cage. The time needed for an animal to obtain the reward (runtime) is considered inversely proportional to the strength of that specific stimulus (Wakonigg et al., 2003; Ettenberg, 2004). Mice that did not reach the goal-chamber within 90 s were gently guided there. Runtimes were manually recorded by two independent experimenters blinded to genotype. After entering the goal-chamber, the door was closed and the mouse was confined there for 2 min, while the cue-light was on. The runway was cleaned with a surface disinfectant between each run.

For cocaine conditioned runway, mice underwent intravenous catheterization minimum 48 h prior to the test. Catheters were flushed daily with 100 μl heparin to ensure proper flow. PE-tubing connected to a 300 μl syringe was attached to the catheter-outlet on the mouse’s back. Mice were acclimatized to the tubing for 10 min prior to the test. The cocaine-enforced runway followed the identical protocol as the food reward-enforced runway, with the exception that the inter-trial-interval was 15 min. During which mice spent 5 min in the goal-box followed by 10 min in a separate resting-chamber. Upon entrance into the goal-box, mice received 0.3 mg/kg cocaine over 6 s with cue-light on. Following the runway-test, 100 mg/kg pentobarbital was injected to control for catheter patency and killing the animals.

### Operant self-administration

For operant self-administration we used standard self-administration chambers produced by medAssociates Inc. The boxes were equipped with two nose poke holes on one wall and a food tray on the opposite. For cocaine self-administration, mice underwent intravenous catheterization minimum 48 h prior to the test. Catheters were flushed daily with 100 μl heparin to ensure proper flow. Mice were allowed to nose poke for 0.15 mg/kg cocaine on a fixed-ratio 1 (FR1) schedule for 2 h daily for a maximum of 10 days. The total amount of infusions was limited to 45 per session. A correct nose poke resulted in a cocaine infusion over 2 s followed by a 20 s time-out. Nose pokes during this period were counted, but not rewarded. Nose-pokes to the incorrect hole had no consequences. To pass criterion, mice had to have ≥75% correct responses over two consecutive days. Active lever presses and rewards are displayed as averages over both sessions in the respective graphs. Upon reaching criterion or after 10 days, 100 mg/kg pentobarbital was injected to control for catheter patency and killing the animals.

The food self-administration paradigm (sugar pellets) followed the identical procedure as the cocaine self-administration, but with the following amendment: Mice were accustomed to the sugar pellets over 2 days prior to the start of the self-administration and were food restricted for 4 h prior to each training session.

### Virus generation and stereotaxic injections

The viral vectors used for Cre-induced deletion of α7 nAChRs were made as previously described (Fritz et al., 2016). For all stereotaxic surgeries, mice were anesthetized with 5% isoflurane induction, placed in the stereotaxic frame (Leica Biosystems) and maintained at 1.0–1.5% isoflurane during surgery. AAV5 viral vectors with or without (control) a Cre-recombinase construct were bilaterally injected at a rate of 100 nl/min into NAc using a gastight Hamilton Neuros syringe (33G); AAV5-hSyn-Cre at a titer concentration of 2,40E+14 gc/ml (Vector Unit, Lund University). AAV5 (250 nl per injection site) for Cre-dependent deletion was delivered in the NAc (AP, +1.1; ML, ±1.2; DV, −4.4). The injection needle was left in place for 10 min after injection to ensure proper diffusion. Animals received analgesic treatment during the first 48 h after surgery with 25 mg/kg buprenorphine (Temgesic®, RB Pharmaceuticals). Behavioral experiments were not started until 3 weeks after the viral injections. Following the behavioral experiments, construct-expression and injection-site location were validated with fluorescent immunohistochemistry. Only results from animals with correct injection-placement and expression were included in the manuscript.

### Quantitative PCR

Brain tissue was used for qPCR analysis. One experimental group of mice received an acute i.p. injection of 15 mg/kg cocaine or saline with the purpose of exploring changes immediate early gene expression; 90 min after the injection, these mice were killed by asphyxiation with CO2. A coronal section of 2 mm, posterior to the olfactory bulb, was removed using a brain matrix in ice cold miliQ-water and the caudate putamen was micro dissected. These samples were immediately frozen on dry ice. Another group of experimental mice were sacrificed by asphyxiation with CO2 followed by cervical dislocation with the aim of exploring Chrna7 expression after conditional knockout. The striatum was dissected, preserved in RNAlater (Qiagen; Hilden, Germany) and stored at 20°C for subsequent analysis. All samples were homogenized in lysis buffer using a TissueLyser (QIAGEN) for 2 min at 20 Hz. RNA extraction was performed with the RNeasy Lipid Tissue Kit (Qiagen). cDNA was synthesized with High Capacity cDNA Reverse Transcription Kit (Applied Biosystems). Quantitative PCR was performed with a Real-Time 7,500 or 7,900 Fast apparatus (Applied Biosystems), using TaqMan Gene Expression Master Mix and TaqMan assay targeting *cFos* (Mm00487425_m1), *FosB* (Mm00500401_m1), *Arc* (Mm01204954_g1), *Egr1* (Mm00656724_m1), *Egr2* (Mm00456650_m1), *cJun* (Mm00495062_s1) or Chrna7 (Mm01312230_m1). *GAPDH* (Mm99999915_g1) was used as endogenous control. Relative quantification was done by the ΔΔCT method.

### Immunohistochemistry

Brains were collected after intracardial perfusion, fast after carbon dioxide asphyxiation, with saline and 4% paraformaldehyde (PFA) in PBS (pH 7.4). The brains were post-fixed for 4 h in 4% PFA and subsequently cryoprotected in 30% sucrose PBS solution overnight. Coronal sections (40 μm) were cut on freezing microtome or cryostat, collected in cold cryoprotectant buffer (0.1 M phosphate buffer, 30% ethylene glycol, 20% glycerol), and stored at −20°C until further use. For immunofluorescent labeling, free-floating sections were washed in PBS, incubated in blocking solution (1% BSA and 0.3% Triton X-100 in PBS), and subsequently incubated with primary antibodies: mouse anti-dopamine-and-cAMP-regulated neuronal phosphoprotein [DARPP32; BD Biosciences, cat# 611520; RRID:AB_398980] 1:1,000, goat anti-choline-acetyltransferase [ChAT; Millipore, cat# AB144; RRID:AB_90650] 1:500 or mouse anti-Cre-recombinase [Cre, Millipore, cat# MAB3120; RRID:AB_2085748] 1:1,000. The following day, the sections were washed and incubated with secondary antibodies: AlexaFlour568 anti-mouse 1:1,000, AlexaFlour568 anti-goat 1:500 or AlexaFlour488 anti-mouse 1:1,000 [Invitrogen] in blocking solution for 2 h. The sections were then washed and mounted on object glasses with VECTASHIELD Hard-Set Antifade Mounting Medium Reagent (Vector Laboratories). Sections were analyzed using a Zeiss Axio Observer Z1 fluorescence microscope connected to a Zeiss LSM 700 confocal unit, Axio Imager 2, with 405, 488, 555, and 639 nm diode lasers, filters 568–1,000, using a Plan-Apochromat 20x/0.8 M27.

### Statistics

Results are illustrated as mean ± SEM. When comparing more than 2 groups with comparable variances, one- or two-way ANOVA was used, followed by *post hoc* analysis with Bonferroni’s multiple comparison tests to evaluate pairwise group differences. In cases of comparisons between two groups an unpaired Student’s t-test was applied. *p* < 0.05 was considered statistically significant. The program GraphPad Prism 6 ® was used for statistical analysis. A tabular overview of statistical results can be found in Supplementary Tables S1-S3.

## Results

### Global deletion of α7 nAChRs impairs cocaine reward learning in conditioned place preference, operant runway and self-administration

**Figure 1:**
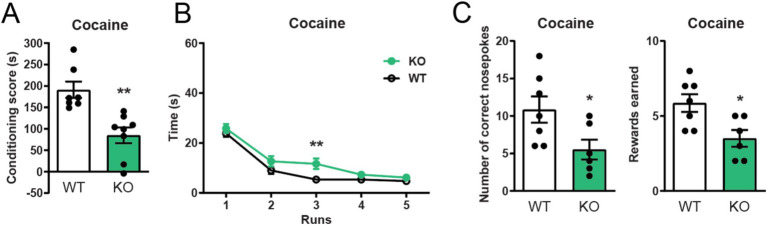
α7 nAChRs are mediators of cocaine reward learning. α7 nAChR −/− mice show a blunted cocaine-seeking response in the conditioned place preference test (*n* = 7 WT, *n* = 8 −/−) **(A)**. α7 nAChR deletion also affects operant responding to cocaine, as α7 nAChR −/− mice have a delay in acquiring the operant runway task to i.v. cocaine (*n* = 8 WT, *n* = 6 −/−) **(B)** and perform fewer correct nose-pokes and self-administer less cocaine (*n* = 7 WT, *n* = 6 −/−) in comparisons to their wild-type littermates **(C)**. Results are displayed as mean ± SEM.*p < 0.05 Student’s unpaired t-test **(A,C)** and **p < 0.01 two-way ANOVA (group factor) followed by Bonferroni’s *post hoc* test **(B)**.

To investigate if constitutive deletion of α7 nAChRs impacts cocaine related behaviors, we started out by studying their role in cocaine-enforced Pavlovian and Operant responding. We found that α7 nAChR −/− mice spent less time in the cocaine-associated compartment in a conditioned place preference paradigm than control mice (Figure 1A). In line with this finding, α7 nAChRs −/− mice also displayed an attenuated motivation to obtain intravenous cocaine infusions as measured by a significantly slower decline in runtime in an operant runway paradigm (Figure 1B, run 3). These findings were further supported, as mice lacking the α7 nAChRs administered significantly less cocaine infusions than controls (Figure 1C) Importantly, no significant differences in number of nose-pokes to the inactive nose-hole were observed between controls and knockout animals (Supplementary Figure S1A).

### α7 nAChRs do not affect acute cocaine locomotor effects or locomotor sensitization

**Figure 2:**
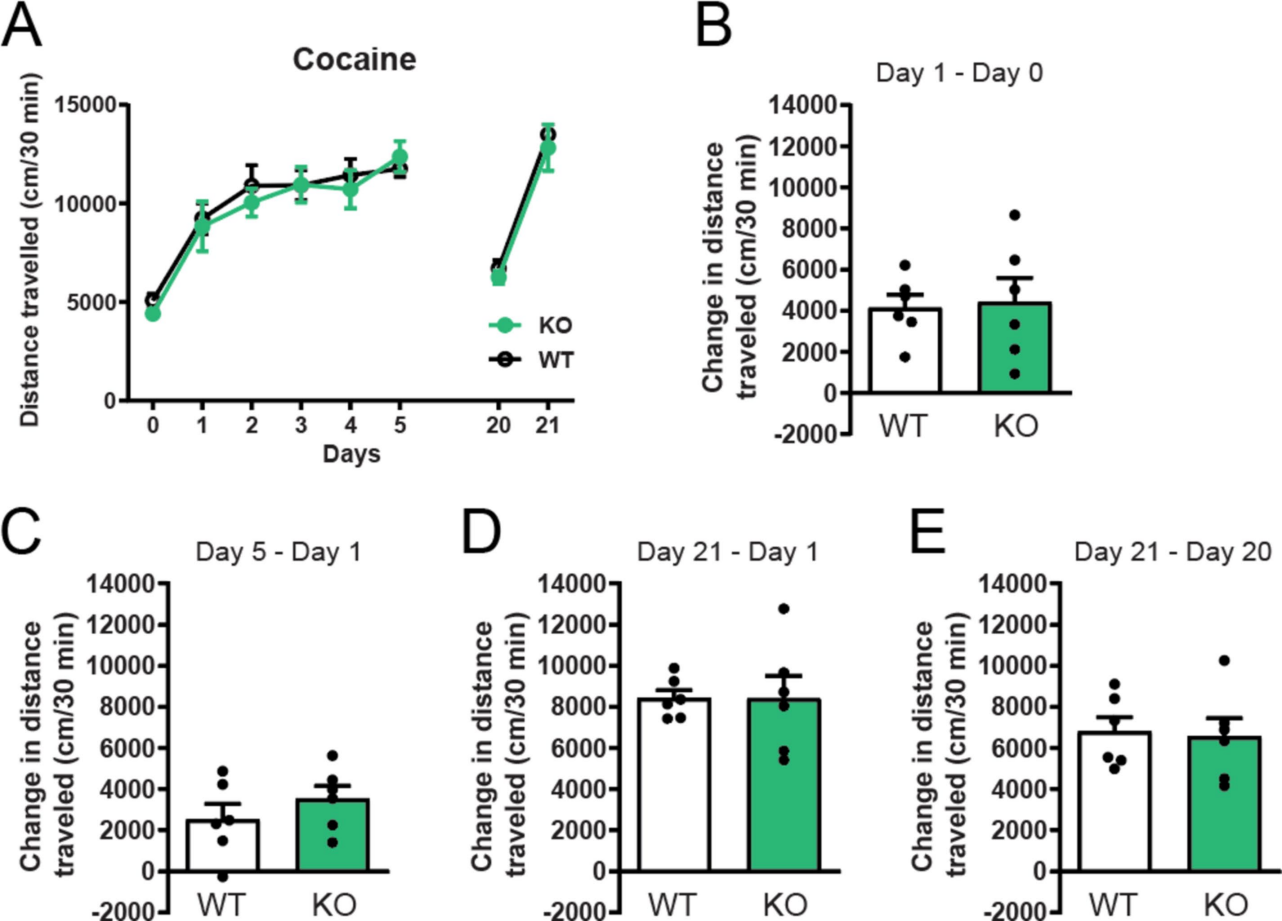
Deletion of α7 nAChRs does not affect cocaine induced locomotion, locomotor sensitization or conditioned sensitization. α7 nAChR −/− mice increased their locomotor response to repeated 15 mg/kg cocaine injections i.p. in the same manner as wildtype littermate controls (*n* = 6 WT, *n* = 6 −/−) **(A)**. No significant differences were observed between wildtype and α7 nAChR −/− mice in acute cocaine-induced locomotion **(B)**, locomotor sensitization **(C)** day 5–1 and **(D)** day 21–1 or conditioned sensitization **(E)** day 21–20. Results are displayed as mean ± SEM. No significant differences were found after analysis with two-way ANOVA followed by Bonferroni’s *post hoc* test **(A)** or Student’s unpaired *t*-test **(B–E)**, *p* > 0.05.

To further explore the role of constitutive deletion of α7 nAChRs receptors for cocaine sensitization, we studied their role in cocaine-induced locomotor effects. Both α7 nAChRs −/− mice and their wild-type (WT) littermates significantly increased their locomotor response to repeated intraperitoneal injections of 15 mg/kg cocaine (Figure 2A). However, no statistical differences were observed between control and knockout mice in baseline locomotion and acute cocaine-induced locomotor responses (Figures 2A,B: day 1 acute cocaine i.p.—day 0 saline i.p.). In line with this, global α7 nAChRs deletion did not affect locomotion after repeated cocaine administration (Figures 2A,C: day 5 cocaine i.p.—day 1 acute cocaine i.p.), nor did it affect behavioral sensitization (Figures 2A,D: day 21 cocaine i.p.—day 1 acute cocaine i.p) and locomotor sensitization (Figures 2A,E: day 21 cocaine i.p.—day 20 no cocaine) compared to control WT animals.

### Global deletion of α7 nAChRs do not affect food-enforced responding

**Figure 3:**
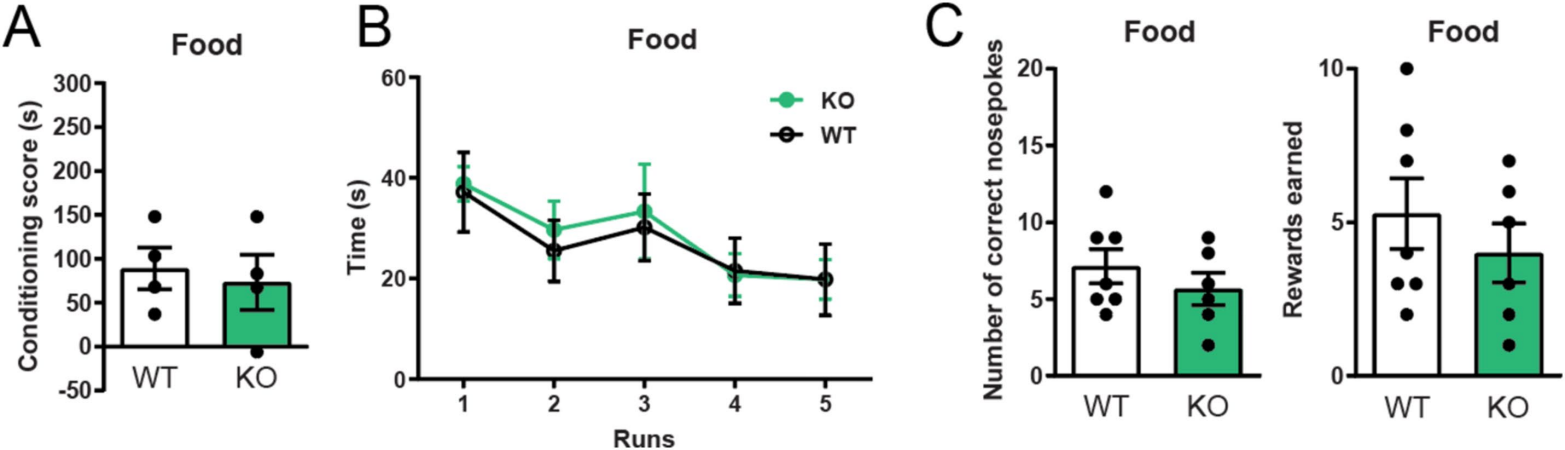
Global deletion of α7 nAChRs does not affect food reward learning Conditioned place preference and operant runway responses to Nutella® **(A,B)**, as well as self-administration of sugar pellets **(C)** do not differ between mice lacking α7 nAChR and wild-type controls (*n* = 4–7 WT, *n* = 4–6 −/−). Results are displayed as mean ± SEM. No significant differences were found after analysis with a Student’s unpaired *t*-test **(A,C)** or two-way ANOVA followed by Bonferroni’s *post hoc* test **(B)**, *p* > 0.05.

Since α7 nAChRs −/− mice show impaired cocaine-induced reward learning, we next investigated if their learning induced by natural rewards were similarly affected. We did this by using the same behavioral tests but replacing cocaine with palatable food. Interestingly, α7 nAChRs −/− mice did not divert from WT littermate controls in behavioral reinforcement-tasks rewarded with food. α7 nAChRs −/− mice and control mice learned a conditioned place preference paradigm and an operant runway task to Nutella® equally well (Figures 3A,B); both groups also acquired the same amount of food-rewards in a self-administration paradigm (Figure 3C), without exhibiting any significant differences in nose-pokes to the inactive nose-hole (Supplementary Figure S1B).

### α7 nAChRs on striatal cholinergic interneurons, but not D1R expressing neurons, are responsible for decreased cocaine responding

**Figure 4:**
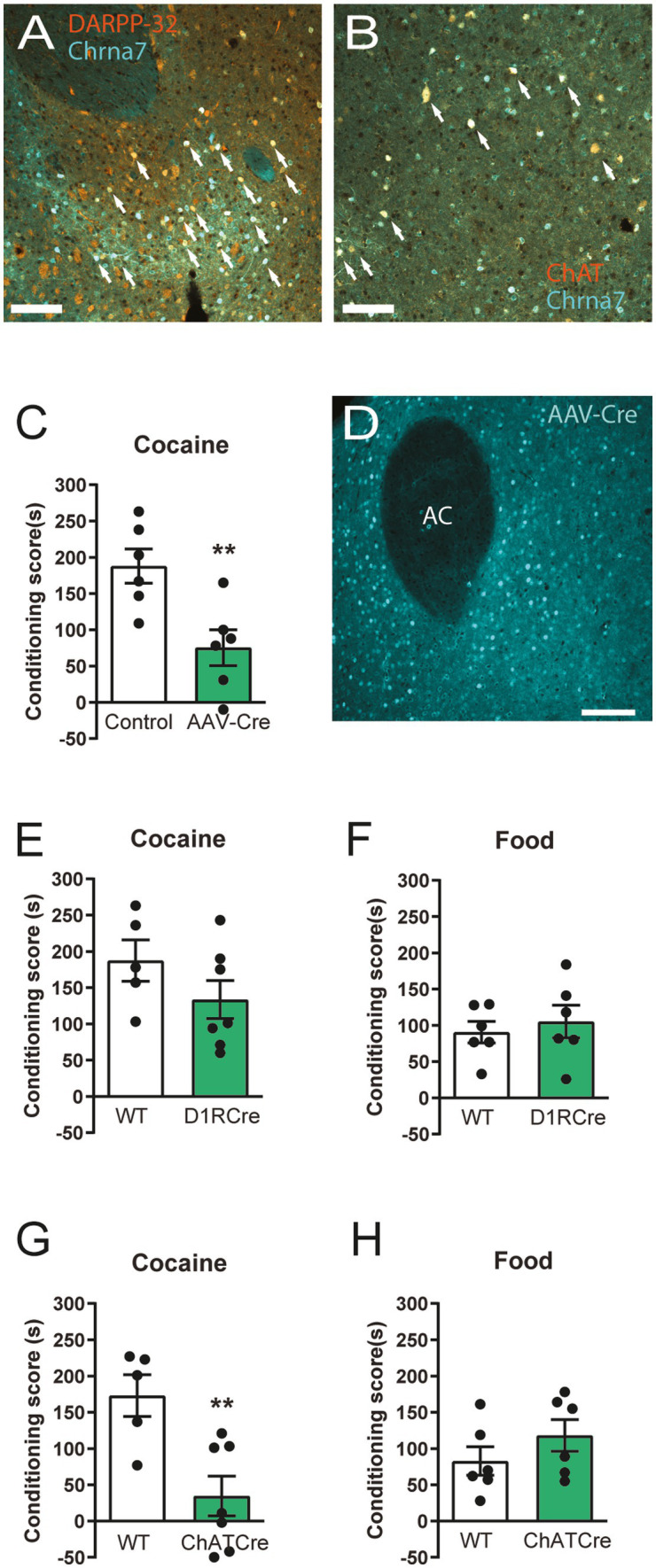
α7 nAChRs on striatal neurons expressing ChAT are mediators of cocaine preference Confocal images (20x magnification, scalebars represent 100 μm) from the striatum showing eGFP expression driven by the Chrna7-promoter in turquoise (pseudo colored) **(A,B)** and medium spiny neuron marker, DARPP-32, staining in orange **(A)** or cholinergic neuron maker (ChAT) staining in orange (pseudo colored) **(B)**. Examples of co-labelling are delineated by arrows. AAV-Cre-recombinase mediated deletion of α7 nAChRs in the NAc decreases cocaine CPP, compared to control AAV-injected animals (*n* = 6 control-virus, *n* = 6 Cre-virus) **(C)**. Representative example (out of *n* = 6) of selective Cre-expression in the striatum of mice injected with the AAV-vector expressing Cre at 20x magnification **(D)**. Scalebar represents 100 μm. Conditional deletion of α7 nAChR in D1R expressing neurons does not elicit a difference, in neither cocaine CPP (*n* = 5 WT, *n* = 7 −/−) **(E)** or food CPP **(F)** (*n* = 6 WT, *n* = 6) −/− paradigms, in mutant mice compared to control animals. Mice with selective deletion of α7 nAChR in ChAT-expressing neurons display a blunted CPP-response to cocaine (*n* = 5 WT, *n* = 7 −/−) **(G)** similar to that of the global α7 nAChR−/− mice (Figure 1A). Conditional deletion of α7 nAChRs from ChAT-expressing neurons does not affect conditioned place preference towards food (*n* = 6 WT, n = 6 −/−) **(H)**. Results are displayed as mean ± SEM. **p < 0.01 Student’s unpaired *t*-test.

As the observed effects from α7 nAChR deletion on cocaine reward learning could arise from striatal neurons, we investigated if α7 nAChRs are expressed on different neuronal populations in the striatum. Using mice with a green fluorescent reporter construct (eGFP) inserted upstream of the α7 nAChR gene (Chrna7), we found that α7 nAChRs are expressed on both DARPP32-expressing medium spiny neurons and ChAT-expressing cholinergic interneurons (Figures 4A,B). Hence, we went on to investigate if the striatum is responsible for the behavioral phenotype of the α7 nAChR −/− mice by stereotaxic injection of an adeno associated viral vector expressing Cre (AAV5-Cre) for selective receptor-deletion in α7 nAChR ^floxed^ animals. Striatal α7 nAChR deletion resulted in a similar blunted cocaine CPP as that of global receptor-deletion in comparison to control mice (Figures 1A, 4C,D; Supplementary Figures S2A-C).

To explore the role of specific striatal α7 nAChR-expressing neuro-populations, such as direct pathway medium spiny neurons (MSNs) or cholinergic projection and interneurons, α7 nAChRs^floxed^ mice were crossed with D1RCre or ChATCre mouse-lines for conditional deletion of the receptors from either dopamine D1R-expressing neurons or cholinergic ChAT-expressing neurons.

We found that deletion of α7 nAChRs from dopaminoceptive D1R expressing neurons did not affect neither food or cocaine CPP (Figures 4E,F), despite reducing α7 nAChR expression levels in the striatum (Supplementary Figures S2D,E; S3A-E). However, mice lacking α7 nAChRs from cholinergic neurons exhibit the same behavioral phenotype as global α7 nAChR −/− mice and mice carrying a deletion of α7 nAChRs in the striatum; as they show decreased Pavlovian responding to cocaine, but intact responding to food reward in comparison to WT controls (Figures 4G,H).

### Cocaine-induced immediate early gene expression in α7 nAChR^floxed^-ChATCre mice

**Figure 5:**
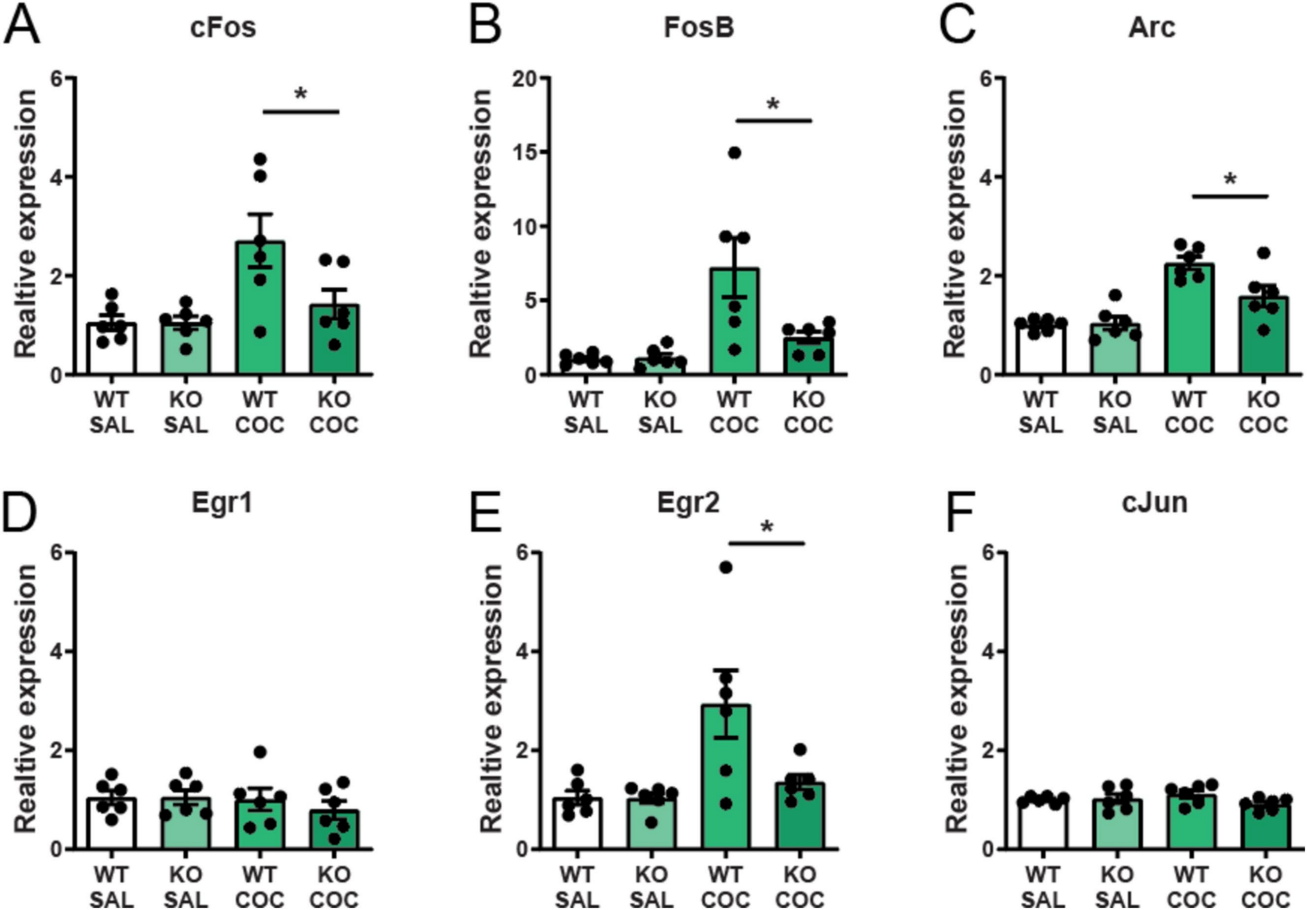
Cocaine-induced immediate early gene expression in the striatum is attenuated in mice lacking α7 nAChRs in cholinergic neurons. The immediate early genes *cfos* **(A)**, *fosB* **(B)**, *arc* **(C)**, and *egr2* **(E)** are upregulated in the striatum of wild-type animals after an acute i.p. injection of 15 mg/kg cocaine, but are significantly attenuated in α7 nAChR^floxed^-ChATCre mice. Expression of *egr1* **(D)** and *cjun* **(F)** was not affected by cocaine (*n* = 6 in all four experimental groups) nor α7 nAChR deletion. Results are displayed as mean ± SEM. *p < 0.05 Two-way ANOVA followed by Bonferroni’s *post hoc* test.

Next, we examined the consequences of α7 nAChR deletion from cholinergic neurons for the striatal expression of immediate early genes (IEGs) involved in neuronal activity, plasticity and motivation (Minatohara et al., 2016). We found that an acute injection of cocaine increased the IEGs *cFos*, *fosB*, *Arc*, and *Egr2* in the striatum of wild-type controls, while induction of these genes were significantly blunted in striatum of mice with selective deletion of α7 nAChRs from Cholinergic neurons (Figures 5A–C,E). There was no induction of *Egr1* and *cJun* after cocaine administration in either control or α7 nAChR ^flox^-ChATCre mice (Figures 5D,F). The decreased IEG response to cocaine reflects neural activity and could explain the neuronal consequences responsible for the observed phenotype in these mice.

## Discussion

In this study, we used a genetic approach to decipher the role of α7 nAChRs expressed on different subpopulations in the striatum for the acquisition of cocaine- and food-enforced learning tasks. We found that global deletion of α7 nAChRs in mice caused a decrease in cocaine reinforcement in CPP, operant runway, and self-administration tasks. In contrast, genetic deletion of α7 nAChRs did not affect acquisition of food-reward in the same tasks. This selective effect of Chrna7 deletion on cocaine-reward over food-reward learning could indicate a cocaine-specific signaling mechanism. In line with this, other studies have found that neither α7 nAChR deletion nor MLA alter behavioral responding to food reward (Young et al., 2011; Hendrickson et al., 2009; Palandri et al., 2021). However, α7 nAChRs have been shown to play a role in reward learning induced by several drugs of abuse: α7 nAChR deletion or selective antagonism reduces reward learning to cannabinoids (Solinas et al., 2007), ethanol (Kamens et al., 2010), nicotine (Markou and Paterson, 2001; Laviolette and van der Kooy, 2003; Walters et al., 2006; Besson et al., 2012) and CPP reinstatement to opioids (Feng et al., 2011; Palandri et al., 2021). Collectively these studies, including ours, indicate that α7 nAChRs play a role in drug reinforcement for several classes of drugs of abuse, without influencing food reward. These properties could make the α7 nAChRs advantageous targets for the treatment of drug addiction.

We did not find any changes in acute cocaine induced locomotion or locomotor sensitization between controls and Chrna7 deletion mice. Locomotor sensitization to drugs of abuse is considered a physiological indicator of synaptic changes in the striatonigral dopaminergic system (Steketee and Kalivas, 2011). The absence of locomotor changes after α7 nAChR deletion may be due to sub-circuitry localization of the receptors on motivational rather than movement circuitry. In line with this, Champtiaux et al. (2006) found that cocaine locomotor sensitization in rats were not affected after MLA injections in VTA and NAc. However, Villégier et al. (2010) found that α7 nAChR deletion decreased acute cocaine induced locomotion over 90 min; the differences between these results and ours could be due to differences in dose (5 vs. 15 mg/kg) and time recorded (90 vs. 30 min as in our case). Further investigations of the α7 nAChR-system are prompted to uncover the foundation for the differences in results obtained between studies.

Previous studies have pointed towards the mesolimbic system, in particular the VTA, for α7 nAChR mediated drug-effects. VTA α7 nAChRs were found to be the central mediators of nicotine reward (Besson et al., 2012; Schilström et al., 2000), and α7 nAChR-expression is upregulated in the NAc after VTA ethanol exposure (Hauser et al., 2021). Here we used a conditional knock-out strategy and found that the α7 nAChRs responsible for cocaine place preference learning are specifically expressed on cholinergic neurons in the striatum; a result strengthened by previous studies showing that optogenetic activation of NAc cholinergic interneurons enhance dopamine release (Cachope et al., 2012) and promote cocaine CPP (Witten et al., 2010). Thus, abolishing the excitatory α7 nAChRs from NAc cholinergic neurons could lead to decreased dopamine release onto MSNs, causing attenuated cocaine reinforcement learning. Interestingly, we found that abolishing cholinergic α7 nAChRs attenuated cocaine induced IEGs, which are considered markers of neural activity. In line with this, several studies have demonstrated that cocaine induction of IEGs in the striatum is dopamine-dependent via D1-receptors (Zhang et al., 2004; Sun et al., 2008; Guan et al., 2009).

There also exist cholinergic brainstem projections from the PPN and LDT to the striatum (Dautan et al., 2014) which could play a role in the observed α7 nAChR effects. Though the PPN and LDT cholinergic projections have been found to modulate dopamine-activity in the VTA (Dautan et al., 2016) this is not the case for their projections to the striatum (Brimblecombe et al., 2018), and the role of brainstem acetyl cholinergic projections to the striatum remains to be explored.

As we also found that α7 nAChRs are expressed on striatal MSNs, and excitability of direct pathway D1R-expressing MSNs are known to cause a reinforcement phenotype (Kravitz et al., 2012), we targeted α7 nAChRs on D1R MSNs. Surprisingly, the D1R-MSN α7 nAChRs did not influence cocaine CPP, which demonstrates how α7 nAChRs may have distinct roles on different circuitry.

We previously demonstrated that genetic ablation of the inhibitory muscarinic M4-receptors on dopaminoceptive D1R-expressing neurons increase cocaine reinforcement behaviors and impulsivity (Klawonn et al., 2018). This study indicated that local cholinergic signaling functions as ‘a break’ on direct pathway D1R MSNs, putting a hold on reward-responding and impulsivity. In the present study, we oppositely find that removing the excitatory α7 nAChRs from cholinergic neurons decreases cocaine reward learning, which would suggest that local cholinergic signaling increases reward learning. This type of dichotomy may arise due to specific circuitry differences, as it has been demonstrated that striatal D1R MSNs have very different effects on reward and aversion dependent on their sub-anatomical localization and their projection targets in the VTA (Yang et al., 2018; Klawonn and Malenka, 2018).

Alternatively, this difference could also be due to the delicate balance of synaptic acetylcholine levels for mediating learning. Acetylcholine is thought to influence reward learning in a bell-shaped manner, where too little or too much transmitter in the synapse is problematic (Grasing, 2016). It is established that elevating acetylcholine levels by acetylcholinesterase inhibitors is only effective at lower doses, whereas higher concentrations impair learning (Braida et al., 1996, 1997; Baldi and Bucherelli, 2005). Several studies on acetylcholine in reward learning have been dichotomous, with results ranging from U-shaped learning curves due to cholinergic receptor agonists, to opposing results from lesion and inhibition studies (Grasing, 2016).

Though it was demonstrated that α7 nAChR−/− mice exhibit procedural learning deficits (Young et al., 2011), we did not observe any such effects in our self-administration and CPP paradigms to food. Consequently, we find it unlikely that an unspecific learning deficit caused the impairments in cocaine-induced reward learning that we observed.

In conclusion, our findings demonstrate that excitatory α7 nAChRs on striatal cholinergic neurons are pivotal for cocaine reward learning, without influencing natural reward. This study supports existing evidence suggesting that the excitability of cholinergic interneurons in the striatum may be key to drugs of abuse responding and provides a piece of the puzzle for understanding the role of cholinergic receptors and their circuits in drug addiction.

## Data Availability

The original contributions presented in the study are included in the article/Supplementary material, further inquiries can be directed to the corresponding author.
