# Supplementary material for: Nicotinic α7 receptors on cholinergic neurons in the striatum mediate cocaine-reinforcement, but not food reward
Source: Front Mol Neurosci. 2025 Jan 21;17:1418686. doi: 10.3389/fnmol.2024.1418686 (PMC11790553; doi:10.3389/fnmol.2024.1418686)
Supplement: Supplementary file 1 [file Supplementary_file_1.docx]

**Supplementary Material**


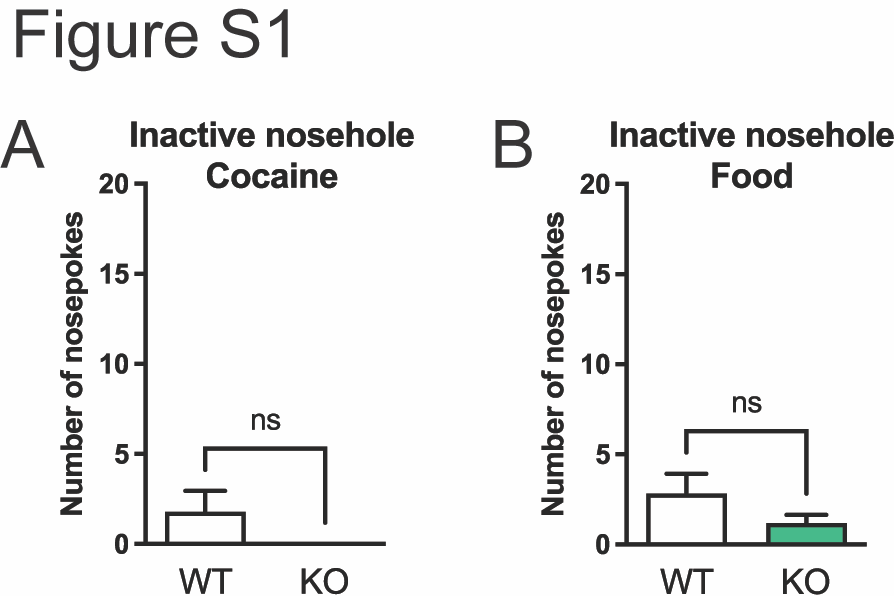


**Figure S1.**  **Inactive nose hole responses of mice with global deletion of α7 nAChRs in cocaine and food self-administration paradigms.**

α7 nAChR -/- mice do not differ from WT controls in responding to the inactive nose hole in cocaine (**A**) (n=4 WT, n=3 -/-) or food **(B)** (n=8 WT, n=6 -/-) self-administration paradigms. Results are displayed as mean ± SEM. Student’s unpaired t-test.

**Table S1. Tabular overview of statistics Unpaired t-tests**

| **Graph** | **Mean**  **(WT vs. KO)** | **SEMs**  **(WT vs. KO)** | **P-value** | **Statistical test** |
| --- | --- | --- | --- | --- |
| **Fig1A: Chrna7 KO Cocaine CPP** | 190.00 vs.  84.75 | ±19.16 vs. ±18.30 | 0.0015 | Unpaired t-test |
| **Fig1C: Chrna7 KO Cocaine Correct Nosepokes** | 10.86 vs.  5.50 | ±1.75 vs. ±1.34 | 0.0375 | Unpaired t-test |
| **Fig1C: Cocaine Rewards earned** | 5.86 vs.  3.50 | ±0.59 vs. ±0.56 | 0.0159 | Unpaired t-test |
| **Fig2B: Cocaine induced locomotion 30min Day 1-Day 0** | 4152 vs.  1169 | ±625.43 vs.  ± 1169.07 | 0.8430 | Unpaired t-test |
| **Fig2C: Cocaine induced locomotion 30min Day 5-Day 1** | 2521 vs.  3539 | ± 757,70 vs.  ± 619,6 | 0.3227 | Unpaired t-test |
| **Fig2D: Cocaine induced locomotion 30min Day 21-Day 1** | 8419 vs.  8408 | ± 398,6 vs. ± 1099 | 0.9928 | Unpaired t-test |
| **Fig2E: Cocaine induced locomotion 30min Day 21-Day 20** | 6794 vs.  6556 | ± 705,2 vs. ± 898,5 | 0.8393 | Unpaired t-test |
| **Fig3A: Food CPP** | 89.00 vs.  73.00 | ±23.84 vs. ±31.63 | 0.7002 | Unpaired t-test |
| **Fig3C: Chrna7 KO Food Correct Nosepokes** | 7.14 vs.  5.67 | ±1.10 vs. ±1.05 | 0.3581 | Unpaired t-test |
| **Fig3C: Chrna7 KO Food Rewards earned** | 5.29 vs. 4.00 | ±1.15 vs. ±0.97 | 0.4193 | Unpaired t-test |
| **Fig4C: Cocaine CPP AAV5-Cre NAc** | 187.80 vs.  75.33 | ±23.60 vs. ±24.54 | 0.0080 | Unpaired t-test |
| **Fig4E: Chrna7-ChatCre Cocaine CPP** | 173.00 vs.  34.57 | ±29.91 vs. ±27.37 | 0.0067 | Unpaired t-test |
| **Fig4F: Chrna7-ChatCre Food CPP** | 82.83 vs.  118.00 | ±19.74 vs. ±21.98 | 0.2614 | Unpaired t-test |
| **Fig4G: Chrna7-D1RCre Cocaine CPP** | 187.40 vs.  133.40 | ±28.47 vs.  ± 26.20 | 0.1996 | Unpaired t-test |
| **Fig4H: Chrna7- D1RCre Food CPP** | 82.83 vs.  118.00 | ±19.74 vs.  ± 21.98 | 0.2614 | Unpaired t-test |
| **FigS1A: Inactive Nosepokes cocaine** | 1.750 vs. 0.000 | ±1.18 vs.  ± 0.00 | 0.2660 | Unpaired t-test |
| **FigS1B: Inactive Nosepokes Food** | 2.813 vs.  1.167 | ±1.11 vs.  ± 0.48 | 0.2501 | Unpaired t-test |

**Table S2. Tabular overview of statistics One way ANOVAs**

| **Graph** | **Mean**  **(WT NaCl vs. KO NaCl WT COC vs. KO COC)** | **SEMs**  **(WT NaCl vs. KO NaCl WT COC vs. KO COC)** | **P-value (ANOVA)** | **F-value (ANOVA)** | **Statistical posthoc test** |
| --- | --- | --- | --- | --- | --- |
| **Fig5A: cFos expression** | 1.053 vs. 1.050  2.709 vs. 1.428 | ±0.15 vs. ±0.13  ±0.53± vs. ±0.29 | 0.0042 | 6.035 | One Way ANOVA, (Bonferroni's multiple comparisons test) |
| **Fig5B: FosB expression** | 1.042 vs. 1.149  7.226 vs. 2.511 | ±0.13 vs. ±0.26  ±1.99 vs. ±0.40 | 0.0011 | 8.020 | One Way ANOVA, Bonferroni's multiple comparisons test |
| **Fig5C: Arc expression** | 1.007 vs. 1.040  2.257 vs. 1.589 | ±0.05 vs. ±0.13  ±0.13 vs. ±0.21 | <0.0001 | 16.67 | One Way ANOVA, (Bonferroni's multiple comparisons test) |
| **Fig5D: Egr1**  **expression** | 1.048 vs. 1.051  1.009 vs. 0.7951 | ±0.14 vs. ±0.72  ±0.52 vs. ±0.58 | 0.7013 | 0.4778 | One Way ANOVA, (Bonferroni's multiple comparisons test) |
| **Fig5E: Egr2 expression** | 1.046 vs.1.035  2.939 vs. 1.356 | ±0.14 vs. ±0.10  ±0.68 vs. ±0.15 | 6.406 | 0.0032 | One Way ANOVA, (Bonferroni's multiple comparisons test) |
| **Fig5F: cJun expression** | 1.002 vs.1.023  1.115 vs. 0.9124 | ±0.03 vs. ±0.10  ±0.08 vs. ±0.05 | 1.452 | 0.2576 | One Way ANOVA, (Bonferroni's multiple comparisons test) |

**Table S3. Tabular overview of statistics Two way ANOVAs**

| **Graph** | **Mean**  **WT vs. KO** | **SEMs**  **Operant runway: WT vs. KO run 1-5**  **Locomotion: WT vs. KO day 0-5, 20-21** | **P-value**  **Time x genotype vs. Time vs.**  **Genotype vs.**  **subject** | **F (DFn, DFd)**  **Time x genotype vs. Time vs.**  **Genotype vs.**  **subject** | **Statistical test** |
| --- | --- | --- | --- | --- | --- |
| **Fig 1B: Chrna7 KO Cocaine Operant Runway** | Run1:  25.83 vs. 23.88  Run2:  12.67 vs. 9.00  Run3:  1.67 vs. 5.375  Run4:  7.333 vs. 5.375  Run5:  6.167 vs. 4.750 | Run1:  ±1.42 vs. ±1.83  Run2:  ±1.43 vs. ±2.08  Run3:  ±0.60 vs. ±2.11  Run4:  ±0.71 vs. ±0.80  Run5:  ±0.65 vs. ±0.70 | 0.1311 vs.  <0.0001 vs.  0.0360 vs.  0.0003 | F(4,48)=1.870  F(4,48)=117.4  F(1,12)=5.577  F(12,48)=3.935 | Two Way ANOVA  (Bonferroni's multiple comparisons test) |
| **Fig2A: Cocaine induced locomotion 30min all days** | Day 0:  5079 vs. 4406  Day 1:  9232 vs. 8828  Day 2:  10899 vs. 10045  Day 3:  10899 vs. 10939  Day 4:  10899 vs. 10717  Day 5:  10899 vs. 12367  Day 20:  6704 vs. 6258  Day 21:  13498 vs. 12814 | Day 0:  ±337.36 vs. ±226.97  Day 1:  ±790.55 vs. ±1263.02  Day 2:  ±1048.30 vs. ±708.16  Day 3:  ±766.69 vs. ±900.22  Day 4:  ±840.11 vs. ±965.18  Day 5:  ±411.09vs. ±784.57  Day 20:  ±410.10vs. ±327.62  Day 21:  ±534.07 vs. ±1185.62 | \| 0.8909 \| \| --- \| \| <0.0001 \| \| 0.6523 \| \| <0.0001 \| | \| F(7,70)=0.4133 \| \| --- \| \| F(7,70)=56.37 \| \| F(1,10)=0.2157 \| \| F(10,70)=9.947 \| | Two Way ANOVA  (Bonferroni's multiple comparisons test) |
| **Fig 3B: Chrna7 KO Food Operant Runway** | Run1:  38.83 vs. 37.17  Run2:  29.67 vs. 25.50  Run3:  33.33 vs. 30.17  Run4:  20.67 vs. 21.50  Run5:  19.83 vs. 19.83 | Run1:  ±7.90 vs. ±3.40  Run2:  ±6.14 vs. ±5.75  Run3:  ±6.60 vs. ±9.44  Run4:  ±6.40 vs. ±4.22  Run5:  ±7.09 vs. ±3.94 | \| 0.9796 \| \| --- \| \| 0.0012 \| \| 0.8175 \| \| <0.0001 \| | \| F(4,40)=0.1066 \| \| --- \| \| F(4,40)=5.551 \| \| F(1,10)=0.05611 \| \| F(10,40)=5.784 \| | Two Way ANOVA  (Bonferroni's multiple comparisons test) |


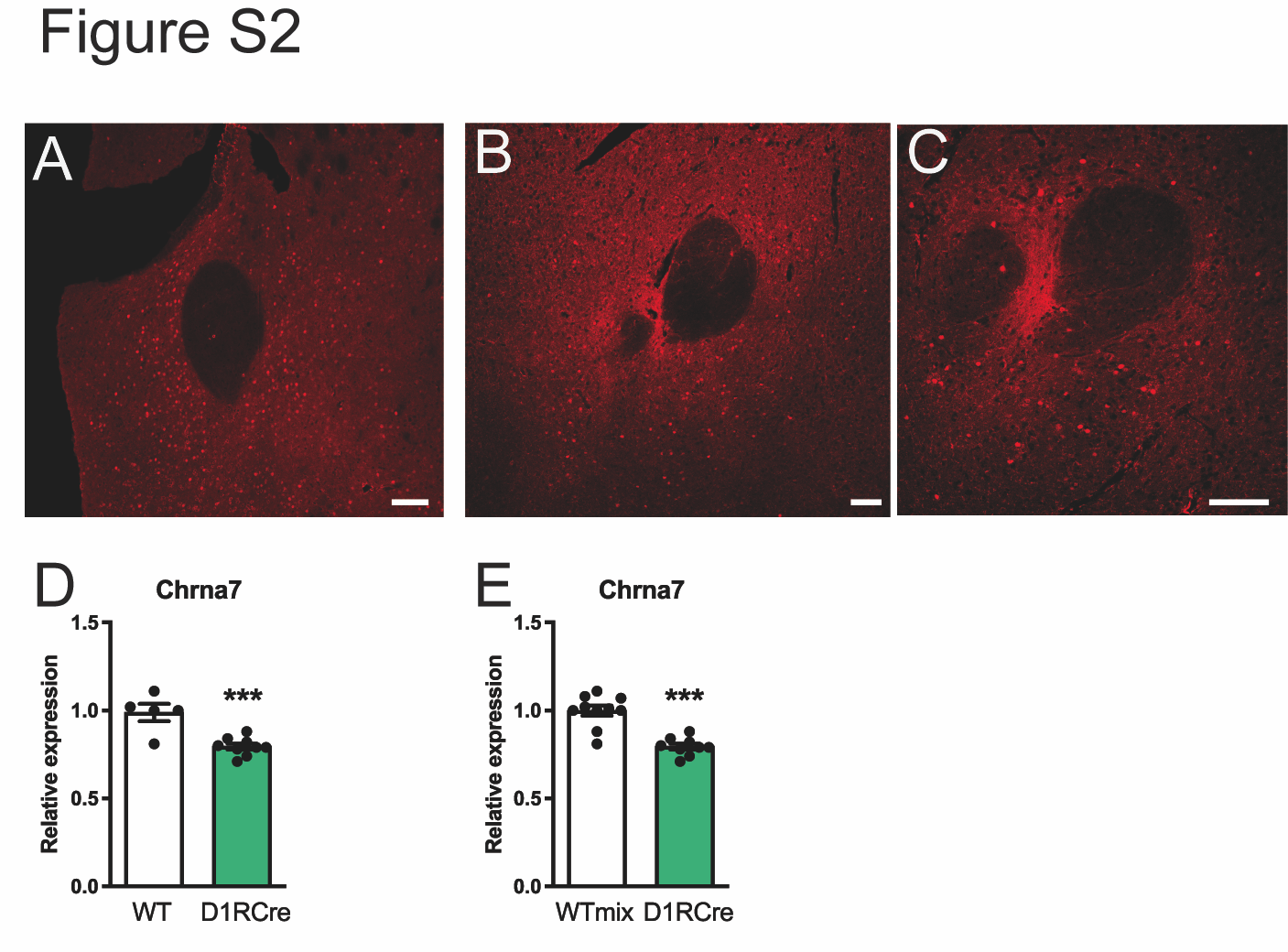


**Figure S2.**  **Representative micrograph examples of Cre-expression in the Nucleus Accumbens of Chrna7 floxed mice**

Representative examples of selective Cre-expression in the NAc of three individual mice injected with the AAV-vector expressing Cre **(A-C)**. 20x images, scalebars represent 100 µm. Conditional deletion of α7 nAChRs from D1R-expressing neurons resulted in a significant reduction of Chrna7R expression (n=5 WT - Cre-negative loxP-positive; n=9 Chrna7R-D1Cre KOs) **(D)** and (WTmix, a mix of Cre-negative and flox-negative controls n= 10; n=9 Chrna7R-D1Cre KOs) **(E)**. Results are displayed as mean ± SEM. ***p < 0.001 Student’s unpaired t-test.


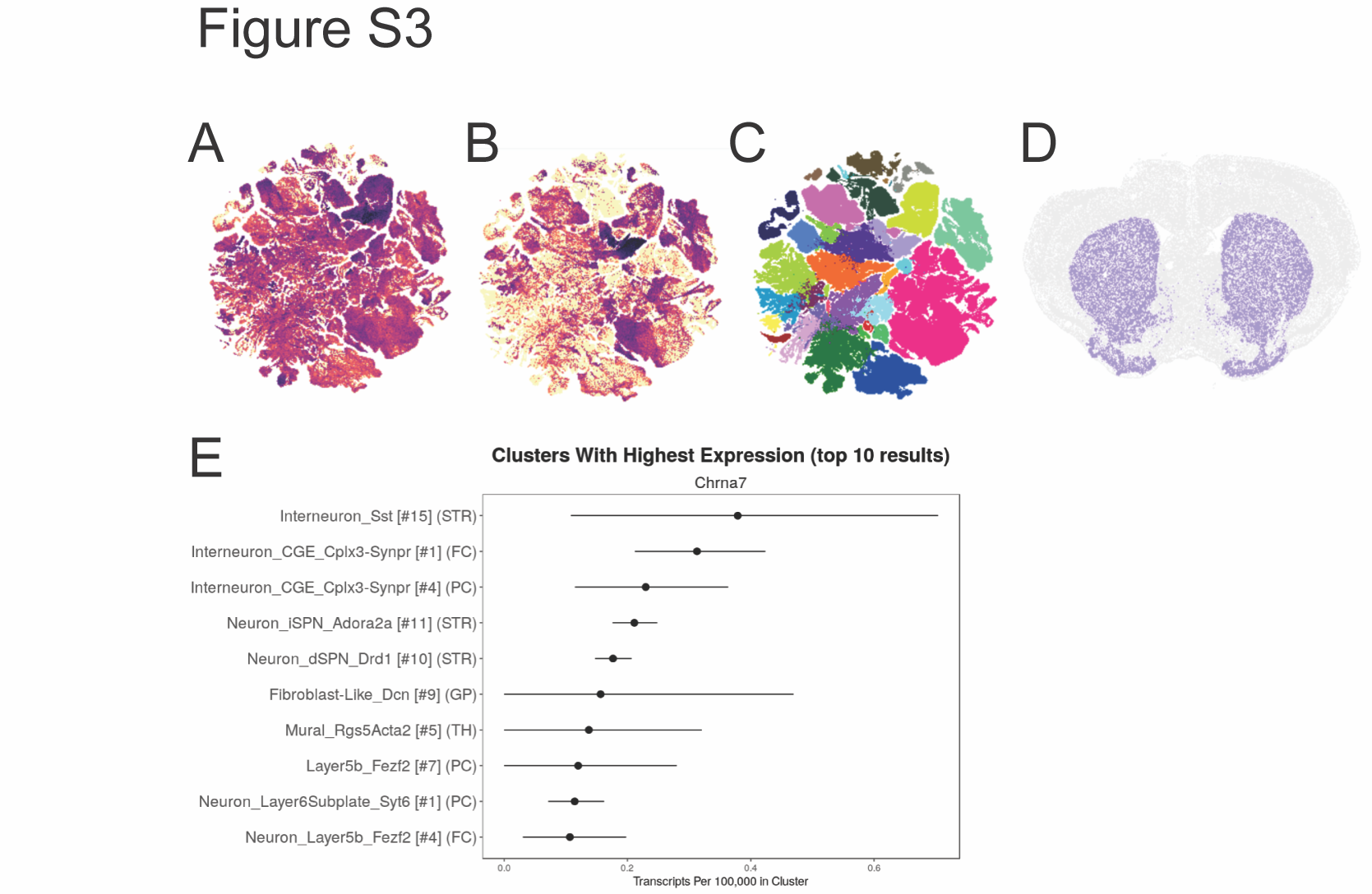


**Figure S3.**  **Analysis of Chrna7 expression in striatal medium spiny neurons**

Analysis of Chrna7 expression using the Allen Brain Cell (ABC) atlas for visualizing multimodal single cell data.  All cell clusters in the whole mouse brain expressing Chrna7 **(A)** and all cell clusters expressing dopamine D1-receptors (D1Rs) **(B)**, color coding of all shared Chrna7 and D1R clusters **(C)**. The corresponding spatially resolved transcriptomics (MERFISH) **(D)** in a coronal mouse brain section demonstrating that the Chrna7-D1R purple cluster (represented in **C**) is in the mouse striatum. Dropviz.org analysis of single cell sequencing data from all mouse brain regions (except hippocampus) **(E)** showing the top ten highest expressing differential neuron clusters.

**Link to Allen Brain Cell Atlas Analysis:**

<https://knowledge.brain-map.org/abcatlas#AQQBQVA4Sk5ONUxZQUJHVk1HS1kxQgACUTFOQ1dXUEc2RlowRE5JWEpCUQADAAQBAQKB3D3qgQikawOFMMAvhF5ibAAFAQFDaHJuYTcAAAYBAAJDaHJuYTcAA34AAAAEAAWAHl43ghp1dgbUNgAIRzRJNEdGSlhKQjlBVFozUFRYMQAJTFZEQkpBVzhCSTVZU1MxUVVCRwAKAAsBbm9uZQACbm9uZQADAQQBAAIjMDAwMDAwAAPIAQAFAQECIzAwMDAwMAADyAEAAAABQVA4Sk5ONUxZQUJHVk1HS1kxQgACUTFOQ1dXUEc2RlowRE5JWEpCUQADAAQBAQKB3D3qgQikawOFMMAvhF5ibAAFAQFEcmQxAAAGAQACRHJkMQADfgAAAAQABYBcSV2C5CVdBsyFAwAIRzRJNEdGSlhKQjlBVFozUFRYMQAJTFZEQkpBVzhCSTVZU1MxUVVCRwAKAAsBbm9uZQACbm9uZQADAQQBAAIjMDAwMDAwAAPIAQAFAQECIzAwMDAwMAADyAEAAAABQVA4Sk5ONUxZQUJHVk1HS1kxQgACUTFOQ1dXUEc2RlowRE5JWEpCUQADBAFGUzAwRFhWMFQ5UjFYOUZKNFFFAAIAAAFRWTVTOEtNTzVITEpVRjBQMDBLAAIAAAExNUJLNDdEQ0lPRjFTTExVVzlQAAIAAAFDQkdDMFUzMFZWOUpQUjYwVEpVAAIAAAQBAQKB3D3qgQikawOFMMAvhF5ibAAFAAYBAQJGUzAwRFhWMFQ5UjFYOUZKNFFFAAN%2BAAAABAAACEc0STRHRkpYSkI5QVRaM1BUWDEACUxWREJKQVc4Qkk1WVNTMVFVQkcACgALAW5vbmUAAm5vbmUAAwEEAQACIzAwMDAwMAADyAEABQEBAiMwMDAwMDAAA8gBAAAAAVpJM1JSMEZYTDNIWVhHVkUyUzUAAkFOSDNVVFM2RFM5MlhCVE9FNEsAAwAEAQACftS0N4Igrf0DgoQbjoF%2Bk90EMk5RVElFN1RBTVA4UFFBSE80UAAFgevbQYEFpwaBWBtJgLxj5AYABwAABQEBQ2NuZDIAAAYBAQJGUzAwRFhWMFQ5UjFYOUZKNFFFAAN%2BAAAABAAACERUVkxFMVlHTlRKUU1XVk1LRVUACUxWREJKQVc4Qkk1WVNTMVFVQkcACgALAW5vbmUAAm5vbmUAAwEEAQACIzAwMDAwMAADyAEABQEBAiMwMDAwMDAAA8gBAAAAAggA>

**Primary publications and datasources:**

Yao et al. (2023) A high-resolution transcriptomic and spatial atlas of cell types in the whole mouse brain. DOI 10.1038/s41586-023-06812-z. [https://www.nature.com/articles/s41586-023-06812-z](https://eur01.safelinks.protection.outlook.com/?url=https%3A%2F%2Fwww.nature.com%2Farticles%2Fs41586-023-06812-z&data=05%7C02%7Cklawonn%40dandrite.au.dk%7C5aeebd2d254a47f3ee1608dc813c4586%7C61fd1d36fecb47cab7d7d0df0370a198%7C1%7C0%7C638527343093638464%7CUnknown%7CTWFpbGZsb3d8eyJWIjoiMC4wLjAwMDAiLCJQIjoiV2luMzIiLCJBTiI6Ik1haWwiLCJXVCI6Mn0%3D%7C0%7C%7C%7C&sdata=1hrsRQXX3Phi%2Fgff2YEBMEOo%2FkVI1i1k%2BOt14yrRz4Q%3D&reserved=0).

10X Dataset: Hongkui Zeng, Zizhen Yao, Cindy van Velthoven, Kimberly A. Smith, Bosiljka Tasic, Changkyu Lee, Jeff Goldy, Anish Bhaswanth Chakka, Thuc Nghi Nguyen, Michael Tieu, Nick Dee,Junitta Guzman, Trangthanh Pham, Amy Torkelson, Rushil Chakrabarty, Tim Dolbeare, Nathan Guilford (2023).

Whole Mouse Brain Transcriptomic Cell Type Atlas - 10x scRNAseq whole brain. [Dataset] Available from [https://assets.nemoarchive.org/dat-qg7n1b0](https://eur01.safelinks.protection.outlook.com/?url=https%3A%2F%2Fassets.nemoarchive.org%2Fdat-qg7n1b0&data=05%7C02%7Cklawonn%40dandrite.au.dk%7C5aeebd2d254a47f3ee1608dc813c4586%7C61fd1d36fecb47cab7d7d0df0370a198%7C1%7C0%7C638527343093650030%7CUnknown%7CTWFpbGZsb3d8eyJWIjoiMC4wLjAwMDAiLCJQIjoiV2luMzIiLCJBTiI6Ik1haWwiLCJXVCI6Mn0%3D%7C0%7C%7C%7C&sdata=kje87nitXpox0%2B%2BlNC%2Bv1bG3wLsifNZ7%2BDaWgnQBbP4%3D&reserved=0). |

MERSCOPE Dataset: Michael Kunst, Delissa McMillen, Jennie Close, Jazmin Campos, Madie Hupp, Naomi Martin, Jocelin Malone, Zoe Maltzer, Augustin Ruiz, Nasmil Valera Cuevas, Brian Long, Jack Waters, Hongkui Zeng. (2023). Whole Mouse Brain Transcriptomic Cell Type Atlas - MERSCOPE v1 [Dataset]. Available from [https://doi.org/10.35077/g.610](https://eur01.safelinks.protection.outlook.com/?url=https%3A%2F%2Fdoi.org%2F10.35077%2Fg.610&data=05%7C02%7Cklawonn%40dandrite.au.dk%7C5aeebd2d254a47f3ee1608dc813c4586%7C61fd1d36fecb47cab7d7d0df0370a198%7C1%7C0%7C638527343093658249%7CUnknown%7CTWFpbGZsb3d8eyJWIjoiMC4wLjAwMDAiLCJQIjoiV2luMzIiLCJBTiI6Ik1haWwiLCJXVCI6Mn0%3D%7C0%7C%7C%7C&sdata=U%2BAcP6jzfU%2FCPraLM4BpsYsBN3DQx57n2fnzSm6s9d8%3D&reserved=0).
